# Changes in cerebral vascular reactivity and structure following prolonged exposure to high altitude in humans

**DOI:** 10.14814/phy2.12647

**Published:** 2015-12-10

**Authors:** Glen E. Foster, Jodie Davies‐Thompson, Paolo B. Dominelli, Manraj K. S. Heran, Joseph Donnelly, Gregory R. duManoir, Philip N. Ainslie, Alexander Rauscher, A. William Sheel

**Affiliations:** ^1^ Centre for Heart, Lung, and Vascular HealthSchool of Health and Exercise ScienceUniversity of British ColumbiaKelownaCanada; ^2^ School of KinesiologyUniversity of British ColumbiaVancouverCanada; ^3^ Department of Ophthalmology and Visual SciencesFaculty of MedicineUniversity of British ColumbiaVancouverCanada; ^4^ Diagnostic and Therapeutic NeuroradiologyVancouver General HospitalUniversity of British ColumbiaVancouverCanada; ^5^ Division of NeurosurgeryDepartment of Clinical NeurosciencesUniversity of CambridgeCambridgeUK; ^6^ Department of RadiologyUBC MRI Research CentreUniversity of British ColumbiaVancouverCanada

**Keywords:** Cerebral atrophy, cerebral vascular reactivity, high altitude

## Abstract

Although high‐altitude exposure can lead to neurocognitive impairment, even upon return to sea level, it remains unclear the extent to which brain volume and regional cerebral vascular reactivity (CVR) are altered following high‐altitude exposure. The purpose of this study was to simultaneously determine the effect of 3 weeks at 5050 m on: (1) structural brain alterations; and (2) regional CVR after returning to sea level for 1 week. Healthy human volunteers (*n* = 6) underwent baseline and follow‐up structural and functional magnetic resonance imaging (MRI) at rest and during a CVR protocol (end‐tidal PCO_2_ reduced by −10, −5 and increased by +5, +10, and +15 mmHg from baseline). CVR maps (% mmHg^−1^) were generated using BOLD MRI and brain volumes were estimated. Following return to sea level, whole‐brain volume and gray matter volume was reduced by 0.4 ± 0.3% (*P* < 0.01) and 2.6 ± 1.0% (*P* < 0.001), respectively; white matter was unchanged. Global gray matter CVR and white matter CVR were unchanged following return to sea level, but CVR was selectively increased (*P* < 0.05) in the brainstem (+30 ± 12%), hippocampus (+12 ± 3%), and thalamus (+10 ± 3%). These changes were the result of improvement and/or reversal of negative CVR to positive CVR in these regions. Three weeks of high‐altitude exposure is reflected in loss of gray matter volume and improvements in negative CVR.

## Introduction

Ascent to high altitude can result in a decrement in neuronal processing, including impairment of arithmetic, memory, language, perception, learning, cognitive flexibility, and psychomotor skills (Wilson et al. 2009; Ainslie et al. 2014). Such decrements in cognitive functioning are implicated in a significant number of deaths above 8000 m (Firth et al. 2008). The underlying pathophysiological mechanisms contributing to neurocognitive deficit when sea‐level residents are exposed to high altitude remain unknown, but may relate to impaired cerebrovascular function and/or neuronal apoptosis (evidenced by loss of gray and/or white matter tissue) even following return to sea level. For example, cognitive deficits are linked to structural brain alterations clinically, and magnetic resonance imaging (MRI) based measurements of regional gray matter volume are a close surrogate for neuronal loss (Teipel et al. 2013). Furthermore, impaired cerebral vascular reactivity can disturb oxygen delivery and is predictive of stroke risk in some clinical scenarios (Yonas et al. 1993).

Although acute exposure to hypoxia (hours to days) can lead to brain swelling (Mórocz et al. 2001; Dubowitz et al. 2009; Rupp et al. 2014), longer term high‐altitude exposure at elevations greater than 5000 m result in neuronal apoptosis in regions of the brain including cortex, hippocampus, and striatum (Maiti et al. 2008). Other structural brain alterations following exposure to high altitude may include an increase in the number of white matter hyperintensities (WMHI) [reviewed in: McGuire et al. (2014)], and gray and white matter atrophy (Garrido et al. 1993; Paola et al. 2008), contributing to a reduction in total brain volume. Hemosiderin deposits (i.e., microhemorrhages) have also been reported in subjects who have experienced high‐altitude cerebral edema (Schommer et al. 2013). Cortical atrophy in humans is linked to impaired cognitive function and has been suggested to occur with chronic high‐altitude exposure (Fayed et al. 2006). Though previous reports (Garrido et al. 1993, 1995; Fayed et al. 2006; Paola et al. 2008; McGuire et al. 2012, 2013; Zhang et al. 2012, 2013; Schommer et al. 2013) have aimed to study changes in brain volume, gray and white matter volume, WMHI, and hemosiderin deposits following high‐altitude exposure the results have been inconsistent. For example, Zhang et al. (Zhang et al. 2012) studied 14 mountaineers before and following ascent to 6206 m and found disruption of white matter fiber integrity but could not replicate the changes in gray matter observed by others (Paola et al. 2008; Yan et al. 2010; Zhang et al. 2013). The lack of agreement between studies could be due to numerous factors including altitude (mild, moderate, or severe), length of stay (acute vs. chronic), time of follow‐up measurement (days to years), or repeated altitude exposure between measurements (impact of repeat exposure and/or intermittent hypoxia). In an attempt to reconcile such differences and build upon previous work, we employed a longitudinal study design, at a moderate altitude often travelled to by trekkers, and made pre and posttest measurements in close succession to limit extraneous factors.

Cerebrovascular reactivity (CVR) to changes in CO_2_ is another important metric of the functional capacity of cerebral blood vessels. Cerebral blood flow increases with hypercapnia and decreases with hypocapnia. A reduction in the magnitude of CVR to changes in CO_2_ has been suggested to indicate impaired vascular function and a reversal of CVR (i.e., negative CVR); such changes are reflective of vascular steal (Sobczyk et al. 2014). Negative CVR and/or vascular steal indicate that in response to a global vasodilatory stimulus, regional blood flow is decreased. There is some evidence in humans that CVR is either unchanged (Willie et al. 2015), reduced (Villien et al. 2013) or enhanced at high altitude (Fan et al. 2010, 2014; Flück et al. 2015). Differences in the method to assess CVR and inaccuracies due to changes in middle cerebral artery (MCA) diameter (Coverdale et al. 2014) may explain between‐study differences. However, there has been little emphasis on the regional CVR response following return to sea level from high altitude where long‐lasting effects of high‐altitude exposure may be observed. Recently, Villien et al. (2013), using an arterial spin‐labeling magnetic resonance imaging (MRI) approach, showed that CVR in the MCA territory is reduced immediately following descent to sea level following 6 days at 4300 m. However, measurements were made immediately following descent from high altitude where acid‐base balance of the blood and cerebral spinal fluid make interpretation difficult. For example, the end‐tidal PCO_2_ differed between baseline and follow‐up measurements by nearly 8 mmHg. The question arises whether or not the reduction in CVR would have remained several days later with normalization of arterial pH and PCO_2_? In addition, although studies show that cortical and anterior/posterior blood flow differences exist in the response of the brain to exposure to normobaric (Binks et al. 2008; Willie et al. 2012; Lewis et al. 2014b) or hypobaric hypoxia (Subudhi et al. 2014), it remains unclear if regional CVR differences assessed by blood oxygen level‐dependent MRI remain upon return to sea level once acid‐base balance has been restored and the lasting effects of high‐altitude exposure can be observed.

The purpose of this investigation was to simultaneously determine the effect of 3 weeks at 5050 m on: (1) structural brain alterations; and (2) regional cerebral vascular reactivity after returning to sea level for 1 week. We hypothesized that 1‐week following exposure to high altitude, (1) overall brain volume would be reduced due to a decrease in gray matter volume; (2) the number of WMHI and/or microhemorrhages would remain unchanged due to the controlled ascent profile; and (3) the CVR would be reduced.

## Materials and Methods

### Ethical approval

All experimental procedures and protocols were approved by the University of British Columbia Clinical Research Ethics Board and conformed to the standards set by the Declaration of Helsinki, and all subjects provided written informed consent prior to participation.

### Participants

Healthy human volunteers (*n* = 7, two female) were studied from a subgroup of participants traveling to the Ev‐K2‐CNR Pyramid Laboratory (5050 m above sea level) as part of the University of British Columbia's International Research Expedition (Foster et al. 2014; Lewis et al. 2014a; Willie et al. 2014). All data presented herein is original and has not been published elsewhere.

All subjects were screened for cardiopulmonary disease by 12‐lead electrocardiogram, pulmonary function testing, resting blood pressure measurements, and a maximal exercise test. Subjects were excluded if they had a history of smoking, were hypertensive (resting systolic >140 mmHg; diastolic >90 mmHg), or had poor pulmonary function based on spirometry measurements [i.e., forced expiratory volume in 1 sec: forced vital capacity (FEV_1_/FVC) ratio less than 0.75]. In addition, all subjects completed a screening form for MRI contraindications. Subjects refrained from caffeine (>12 h), alcohol and exercise (>24 h) prior to experimental measurements.

### Experimental protocol

All subjects attended three experimental testing sessions at sea level. During the first visit, subjects were screened for study inclusion and were familiarized with the breathing circuit and breathing tests that were to be subsequently conducted inside the MRI suite. Visit two and three were identical and involved structural and functional imaging of the brain using MRI at rest and during a CVR test to hypo/hypercapnia. The CVR test involved two, two‐minute steps of hypocapnia [end‐tidal partial pressure of CO_2_ (petCO_2_) reduced by 10 and 5 mmHg respective to baseline by voluntary hyperventilation] and three, two‐minute steps of hypercapnia (petCO_2_ increased by 5, 10, and 15 mmHg respective to baseline during eupnea). During the CO_2_ test the end‐tidal partial pressure of O_2_ (petO_2_) was clamped at 100 mmHg for all trials. This protocol has been used previously to assess CVR by Doppler ultrasound of both intracranial and neck vessels including the study of cerebral blood flow regulation on this expedition (Willie et al. 2015).

Between the second and third testing sessions, subjects were involved in a research expedition to the Ev‐K2‐CNR Pyramid Laboratory. The trek involved 1 week in Kathmandu (elevation: 1400 m) and an 8‐day trek from Lukla (elevation: 2860 m) to the Pyramid Laboratory where the subjects remained for 3 weeks prior to descending to Kathmandu over a 4‐day period. The trekking profile and the anterior/posterior changes in cerebral blood flow (measured using ultrasound methods) during this trek have previously been reported (Willie et al. 2014). The second and third testing session took place in Vancouver at sea level 1 day prior to departure to Nepal and 1 week following return from Nepal. While at the Pyramid Laboratory, the subjects were involved in various research projects aimed at understanding the cerebral, cardiac, and pulmonary response to high altitude (Foster et al. 2014; Willie et al. 2014). During the 8‐day trek to the laboratory, subjects ingested acetazolamide (125 mg three times daily) to aid in acclimatization and minimize the risk and severity of acute mountain sickness. The Lake Louise and environmental symptoms – cerebral symptoms questionnaires were used to assess acute mountain sickness (Sampson et al. 1983). Acetazolamide ingestion was withdrawn 24 h prior to arriving at the pyramid laboratory. A physician was present to manage patient symptoms and conservatively prescribe treatment where necessary.

### Experimental techniques

#### Magnetic resonance imaging

After the subject completed the screening questionnaires for MRI they were instrumented with a facemask and breathing apparatus and placed inside a 3.0 tesla MRI scanner (Philips Achieva) in the supine position. Scans were conducted using an 8‐channel SENSE head coil in the following order: T1‐weighted imaging for anatomical structures and volumetric analyses, multi echo susceptibility‐weighted imaging (SWI)(Denk and Rauscher 2010) to determine microhemorrhages (five echoes, TE_1_ = 6 msec, echo = 6 msec; TR = 34 msec; voxel size = 0.5 × 0.5 × 1 mm^3^), and 3D fluid attenuated inversion recovery (FLAIR) imaging for WMHI (TR = 8000 msec, TI = 2400 msec, TE = 156 msec, voxel size = 1 × 1 × 1 mm^3^). Blood oxygen level‐dependent (BOLD) imaging for CVR to changes in arterial CO_2_ was performed using T2*‐weighted echo planar imaging with 36 interleaved axial slices (TR = 2000 msec, TE = 30 msec, FOV = 240 × 216 mm, 3 mm thickness with 1 mm gap, voxel size = 3 × 3 mm, 128 mm reconstruction matrix, reconstructed voxel size 1.88 × 1.6 mm). FLAIR and SWI were read for WMHI and microhemorrhages by an experienced neuroradiologist (MH) who was blinded to subject and the experimental condition.

#### Respiratory control and measurements

Respiratory and cardiovascular parameters were acquired at 200 Hz using an analog‐to‐digital converter (PL3504, ADinstruments, Colorado Springs) interfaced with a personal computer and analyzed using commercially available software (LabChart, ADinstruments, Colorado Springs). Throughout resting and CO_2_ testing procedures subjects breathed through a facemask and two‐way nonrebreathing valve. Respired gas concentrations were sampled at the mouth and analyzed for PO_2_ and PCO_2_ (model #17620 and #17630, VacuMed, Ventura). Respiratory flow was measured at the mouth using a pneumotachograph (HR 800L, HansRudolph, Shawnee). petO_2_, petCO_2_, inspiratory (VT_I_), and expiratory tidal volume (VT_E_) were determined for each breath online using specifically designed software (Labview 13.0, National Instruments, Austin). Arterial oxyhemoglobin saturation (SpO_2_) was measured by finger pulse oximetry (7500FO, Nonin Medical Inc, Plymouth). petO_2_ and petCO_2_ were controlled by a portable end‐tidal forcing system designed specifically for use in the MRI suite. The end‐tidal gas control system uses independent gas solenoid valves for O_2_, CO_2_, and N_2_ and controls the volume of each gas being delivered to the inspiratory reservoir through a mixing and humidification chamber. petCO_2_ using this gas control system is within 2.1 ± 0.5 mmHg of arterial PCO_2_ at baseline and during hypercapnia (Tymko et al. 2015). Resting parameters were acquired with the subject in the supine position inside the MRI scanner immediately before conducting the CVR test.

### MRI analysis

Two time‐point percentage brain volume change (PBVC) was estimated with SIENA (Smith et al. 2002), part of FSL (FMRIB's Software Library, www.fmrib.ox.ac.uk/fsl) (Smith et al. 2004). SIENA extracts brain and skull images from the two time‐point whole‐head input data and subsequently aligns the two images with each other (Jenkinson et al. 2002; Smith et al. 2002). Next, tissue‐type segmentation is carried out in order to find brain/nonbrain edge points, and then perpendicular edge displacement between the two time points is estimated at these edges (Zhang et al. 2001). Finally, the mean edge displacement is converted into a global estimate of PBVC between the two time points. The global estimate of PBVC between the two time points was assessed statistically by one sample t‐test referenced to zero percent change. Global gray matter and white matter volume were estimated from partial volume estimates from the normalized segmented output from SIENA and were used to generate gray and white matter masks for extraction of CVR data (see below). Paired t‐tests were used to determine if gray and white matter volume differed with exposure to high altitude. Finally, focal gray matter atrophy was analyzed using FSL's optimized voxel‐based morphometry protocol (Smith et al. 2004). A voxel‐wise general linear model was applied using permutation‐based nonparametric testing, correcting for multiple comparisons across space. *z*‐statistic maps were generated and considered significant at a resel‐corrected *P*‐value of 0.05. The resel correction uses a principle similar to Gaussian random field theory to estimate the number of independent samples (resels) within a dataset (Worsley et al. 1998), and is therefore less conservative than a Bonferroni correction.

BOLD data processing was carried out using FEAT (FMRI Expert Analysis Tool, Version 6.0), part of FSL. Registration to individual participants T1‐weighted anatomical image and to standard (MNI) space was carried out using FLIRT (Jenkinson et al. 2002). Motion correction was applied using MCFLIRT (Jenkinson et al. 2002), followed by slice‐timing correction, spatial smoothing (a Gaussian, FWHM 6 mm), and high‐pass temporal filtering (Gaussian‐weighted least‐squares straight line fitting, with sigma = 0.5sec).

Time‐series statistical analysis was carried out using FILM with local autocorrelation correction (Woolrich et al. 2001). petCO_2_ and SpO_2_ were entered as explanatory and nuisance variables, respectively. For each subject, petCO_2_ and petO_2_ were time corrected for sample and circulation delay by cross‐correlation (Poublanc et al. 2013) with whole‐brain BOLD time course prior to modeling and statistical analysis for both baseline and follow‐up conditions. For general linear modeling, SpO_2_ was calculated from petO_2_ based on the equations defined by Severinghaus (Severinghaus 1979) to correct for the circulatory delay. petCO_2_ parameter estimates were converted to percent signal change and CVR maps (% mmHg^−1^) were generated by indexing against the change in petCO_2_.

Average CVR was extracted separately for gray matter, white matter, and anatomical regions of interest from the Harvard‐Oxford Subcortical Structural Atlas. Since areas of negative CVR could influence the overall CVR in each anatomical structure, further analysis was conducted on only those voxels showing a positive CVR and a resel‐corrected *z*‐statistic with *P* < 0.05 from each region. This was then repeated for regions showing a negative CVR. At follow‐up the data were extracted from the same regions to determine how high‐altitude exposure affected regions of negative and/or positive CVR.

### Statistical analysis

A two‐by‐two repeated measures analysis of variance was used to determine the effect of high‐altitude hypoxia on the left and right hemisphere structures. A paired *t*‐test was used for structures such as the brainstem, which was not categorized by hemispheres. A two‐by‐two repeated measures analysis of variance was also used to determine the effect of high‐altitude exposure on areas of positive and negative CVR. In all cases, when significant *F*‐ratios were uncovered, Tukey's HSD post hoc analysis was conducted. Statistical tests were conducted using commercially available software (Statistica V.7.0, Statsoft Inc., Tulsa). Respiratory parameters were compared at rest (paired *t*‐test) and throughout the vascular reactivity test (repeated measures ANOVA). In all cases *P* < 0.05 was considered statistically significant. Values are presented as means ± SE.

## Results

### Subjects

One female subject was excluded from posttesting because she was prematurely evacuated from our high‐altitude laboratory for medical reasons unrelated to experimental testing. Consequently, six subjects are included in the analyses reported here. Six subjects (age = 31.8 ± 3.7 years; BMI = 27.0 ± 3.6 kg m^−2^) completed all experimental sessions including pre‐ and post‐MRI measurements. Subjects had normal resting pulmonary function (FEV_1_/FVC = 83.0 ± 4.9% or 107 ± 5% predicted), cardiovascular health (resting MAP = 89 ± 3 mmHg; HR = 62 ± 11 beats min^−1^) and met the study inclusion/exclusion criteria. During the trek to high altitude and during the first few days at 5050 m, subjects were monitored for acute mountain sickness. All subjects developed symptoms of acute mountain sickness with a Lake Louise acute mountain sickness score >3. However, two male subjects had symptoms necessitating intervention including 2 h of supplemental oxygen (*n* = 1; within 6 h of arrival at 5050 m) and intravenous dexamethasone (*n* = 1; required after 1 week at 5050 m). The remaining subjects experienced mild symptoms of acute mountain sickness that did not warrant treatment.

### Volumetric analyses

Brain volume was reduced by an estimated 0.4 ± 0.3% (*P* < 0.01) following exposure to high altitude. This response ranged from a −0.21 to −1.00% estimated percent brain volume change. Gray matter density, analyzed by voxel‐based morphometry, did not detect altitude induced focal changes. However, global volumetric calculations revealed a reduction in gray matter (−2.6 ± 1.0%; *P* < 0.001) but not white matter (−1.3 ± 0.7%; *P* = 0.14). Table 1 displays the absolute and percent change in gray matter and white matter volume for each subject.

**Table 1:** Gray matter and white matter volumetric analysis and white matter hyperintensities (WMHI) before (Pre) and after (Post) exposure to high altitude for all subjects

Subjects	Gray matter (mL)			White matter (mL)			WMHI (#)	
	Pre	Post	% Change	Pre	Post	% Change	Pre	Post
1	733	718	−2.1	666	652	−2.0	3	6
2	569	544	−4.3	509	490	−3.7	1	1
3	653	631	−3.4	614	623	+1.5	3	4
4	686	670	−2.4	659	659	0.0	38	37
5	701	689	−1.6	643	633	−1.5	–	–
6	667	653	−2.0	601	590	−1.8	0	0
Mean (SE)	668 (23)	651 (25)	−2.6 (0.4)^a^	615 (24)	608 (25)	−1.3 (0.7)		

a *P* < 0.001.

### Cerebral vascular reactivity

**Figure 1:**
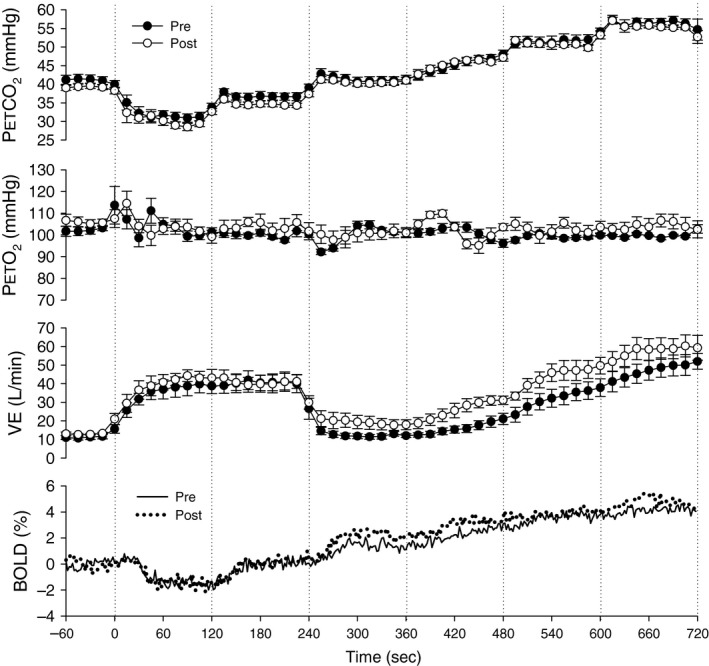
Group mean ventilatory and cerebral vascular response to the vascular reactivity test evoked by stepwise changes in CO_2_ before (PRE) and after (POST) exposure to high‐altitude hypoxia. % BOLD is displayed as the mean BOLD signal for the brainstem for the entire group (*n* = 6) at 2 sec intervals, whereas the remaining data points are displayed as 30 sec mean ± SE for the group (*n* = 6). The vertical dotted lines define each step change in CO_2_. pet CO_2_, end‐tidal partial pressure of carbon dioxide; petO_2_, end‐tidal partial pressure of oxygen; V̇_E_, minute ventilation; % BOLD, the blood oxygen level‐dependent signal expressed as percent change from baseline.

**Figure 2:**
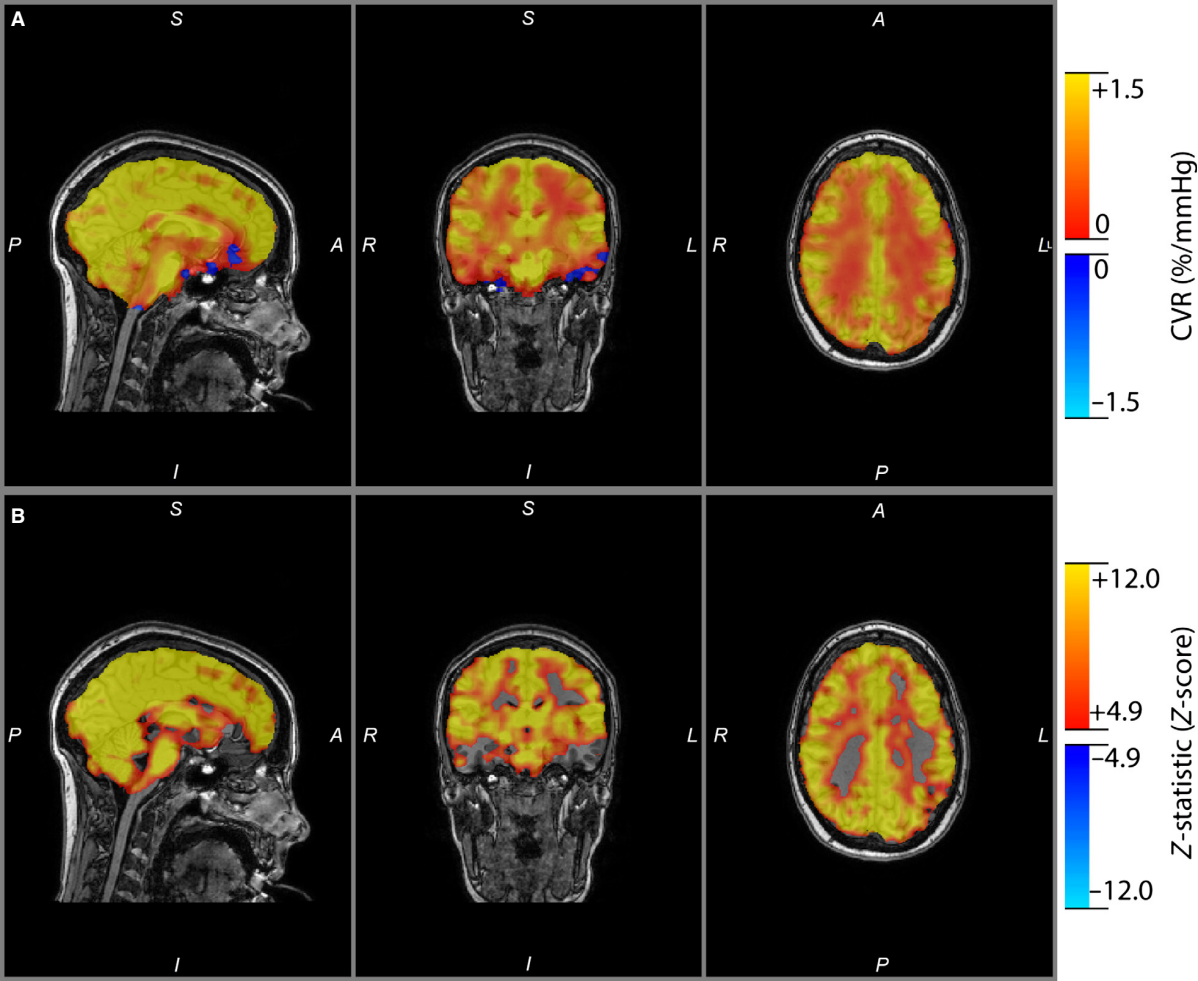
(A) Whole‐brain cerebral vascular reactivity map and (B) *z*‐statistic map resel‐corrected to *P* < 0.05 for one representative subject. The *z*‐statistic provides a measure of how well the general linear model fits the BOLD signal to pet CO_2_ and thus illustrates a strong linear relationship between BOLD and pet CO_2_.

**Figure 3:**
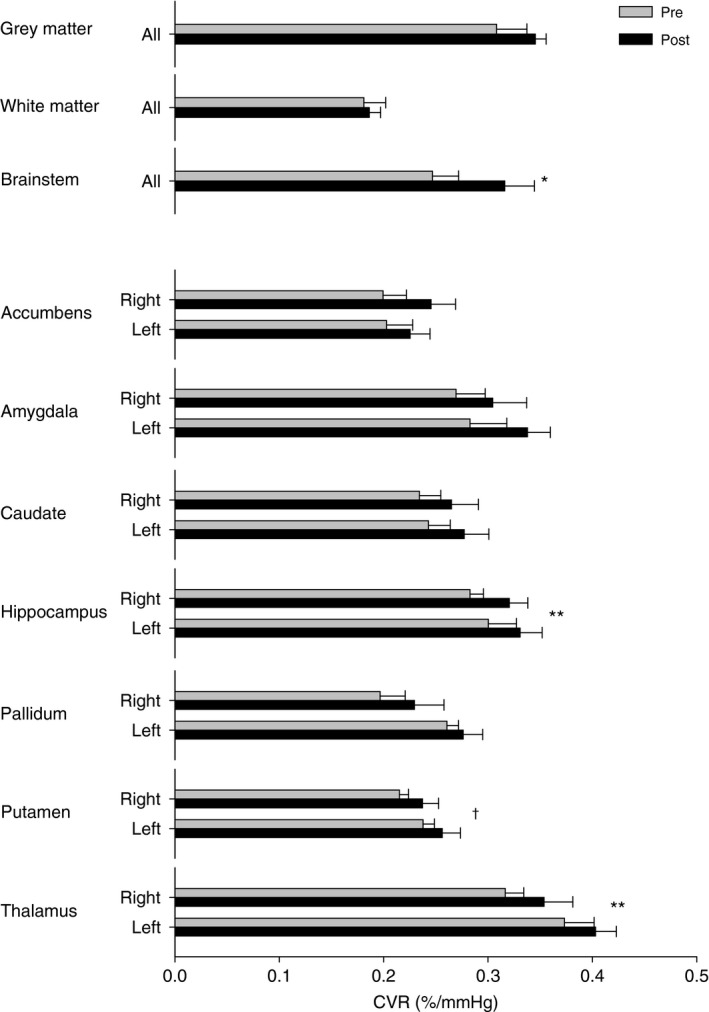
Displays the cerebral vascular reactivity (CVR) of different subcortical structures before (Pre) and after (Post) exposure to high‐altitude hypoxia. Where appropriate structures have been separated into left and right hemispheres. Values are mean ± SE. *P < 0.05 Pre versus Post; *^*^ *P* < 0.01 Pre versus Post; ^†^ *P* < 0.05 Left versus Right.

Resting breathing frequency, tidal volume, minute ventilation, petO_2_, petCO_2_, and SpO_2_ were acquired from the subject inside the MRI scanner (Table 2). Subjects were in a similar resting state with normal respiratory gases. Figure 1 displays the group mean time course of petCO_2_, petO_2_, minute ventilation, and the resultant %BOLD response of the brainstem during the CVR measurement before and following exposure to high‐altitude hypoxia. The steps in petCO_2_ were the same between conditions and petO_2_ was held constant throughout both tests. Figure 2 displays the resultant CVR and *z*‐statistic maps for one representative subject at follow‐up. The number of significant voxels in the *z*‐statistic map suggests that over the range of petCO_2_ studied the BOLD response is suitably linear. It can also be noted that gray matter responds to a much greater degree than white matter (see Fig. 2). Table 3 provides an individual account of CVR for white and gray matter and highlights the nearly twofold difference (*P* < 0.01) in gray and white matter CVR (see also Fig. 3).

**Table 2:** Subject baseline characteristics before (pre) and after (Post) exposure to high altitude

	Pre	Post	P
F_b_, breaths min^−1^	12.6 (1.6)	13.9 (1.0)	0.39
V_T_, L	1.0 (0.1)	0.94 (0.1)	0.54
V̇_E_, L min^−1^	11.0 (1.1)	12.7 (0.5)	0.18
petO_2_, mmHg	101.8 (1.8)	105.6 (2.0)	0.27
petCO_2_, mmHg	41.4 (1.2)	39.5 (0.7)	0.13
SpO_2_, %	96.4 (0.3)	97.1 (0.3)	0.10

Values are mean (SE). F_b_, breathing frequency; V_T_, tidal volume; V̇_E_, minute ventilation; petO_2_, end‐tidal partial pressure of oxygen; petCO_2_, end‐tidal partial pressure of carbon dioxide; SpO_2_, arterial oxyhemoglobin saturation.

**Table 3:** Gray matter and white matter cerebral vascular reactivity before (Pre) and after (Post) exposure to high altitude for all subjects

Subjects	Gray matter CVR (% mmHg^−1^)			White Matter CVR (% mmHg^−1^)		
	Pre	Post	% Change	Pre	Post	% Change
1	0.30	0.39	+31.5	0.27	0.20	−25.8
2	0.34	0.42	+21.3	0.19	0.21	+8.8
3	0.27	0.31	+15.2	0.15	0.18	+20.6
4	0.31	0.23	−24.3	0.12	0.16	+28.9
5	0.33	0.41	+24.8	0.19	0.22	+16.5
6	0.31	0.32	+3.1	0.17	0.15	−7.1
Mean (SE)	0.31^a^ (0.01)	0.35^a^ (0.03)	+11.9 (8.24)	0.18 (0.02)	0.19 (0.01)	+6.98 (8.24)

a *P* < 0.01 compared to respective white matter CVR.

Following exposure to high‐altitude hypoxia, individual subjects displayed focal increases in CVR. However, these areas did not colocalize between subjects. To determine regional changes in CVR, masks were created for all structures contained within the Harvard‐Oxford Subcortical Structural Atlas. In addition, gray and white matter masks were created. Figure 3 shows the average CVR for all subjects from each of these regions. CVR was greatest in gray matter and lowest in white matter. Generally, the brainstem, amygdala, hippocampus, and the thalamus had the highest CVR compared to other structures. There were no differences between the CVR in left and right hemispheres except for the putamen that had a greater CVR in the left hemisphere (*P* < 0.05).

**Figure 4:**
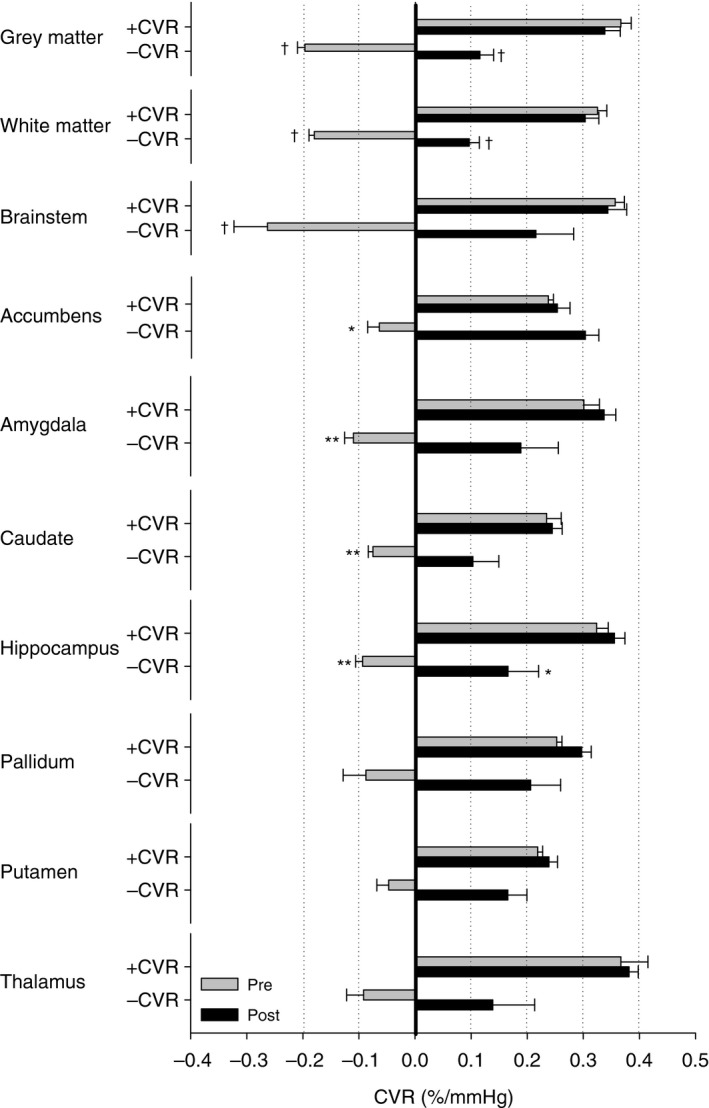
Displays the cerebral vascular reactivity (CVR) of different subcortical structures based on pretest positive CVR (+CVR) and negative CVR (−CVR) voxel masks. Negative CVR in the pretest was often reversed by exposure to high altitude. Few subjects demonstrated negative reactivity when only considering voxels with a significant *z*‐statistic (*P* < 0.05) in the pallidum, putamen, and thalamus limiting statistical power. In all other structures, negative CVR was recorded in all subjects. Statistical symbols if marked on –CVR pre are statistically different from both +CVR and −CVR post. Alternatively, if statistical symbol is marked on −CVR post the difference is compared to +CVR. *P < 0.05; **P < 0.01; ^†^ *P* < 0.001.

Following 3 weeks at high altitude, CVR was increased in the brainstem, hippocampus, and thalamus. After segmenting the structures into significant (i.e., *P* < 0.05) voxels of positive and negative reactivity during the pretest, we determined that this was the result of an improvement and/or reversal of negative reactivity to positive (Fig. 4). As a result, regions of positive reactivity during the pretest maintained their reactivity, whereas regions of negative reactivity during the pretest were substantially reduced in size and became positive on average during the posttest. This result was most notable for gray matter, white matter, brainstem, nucleus accumbens, caudate, and hippocampus. The pallidum, putamen, and thalamus often did not have any significant voxels of negative reactivity and hence statistical testing could not be conducted for these regions.

**Figure 5:**
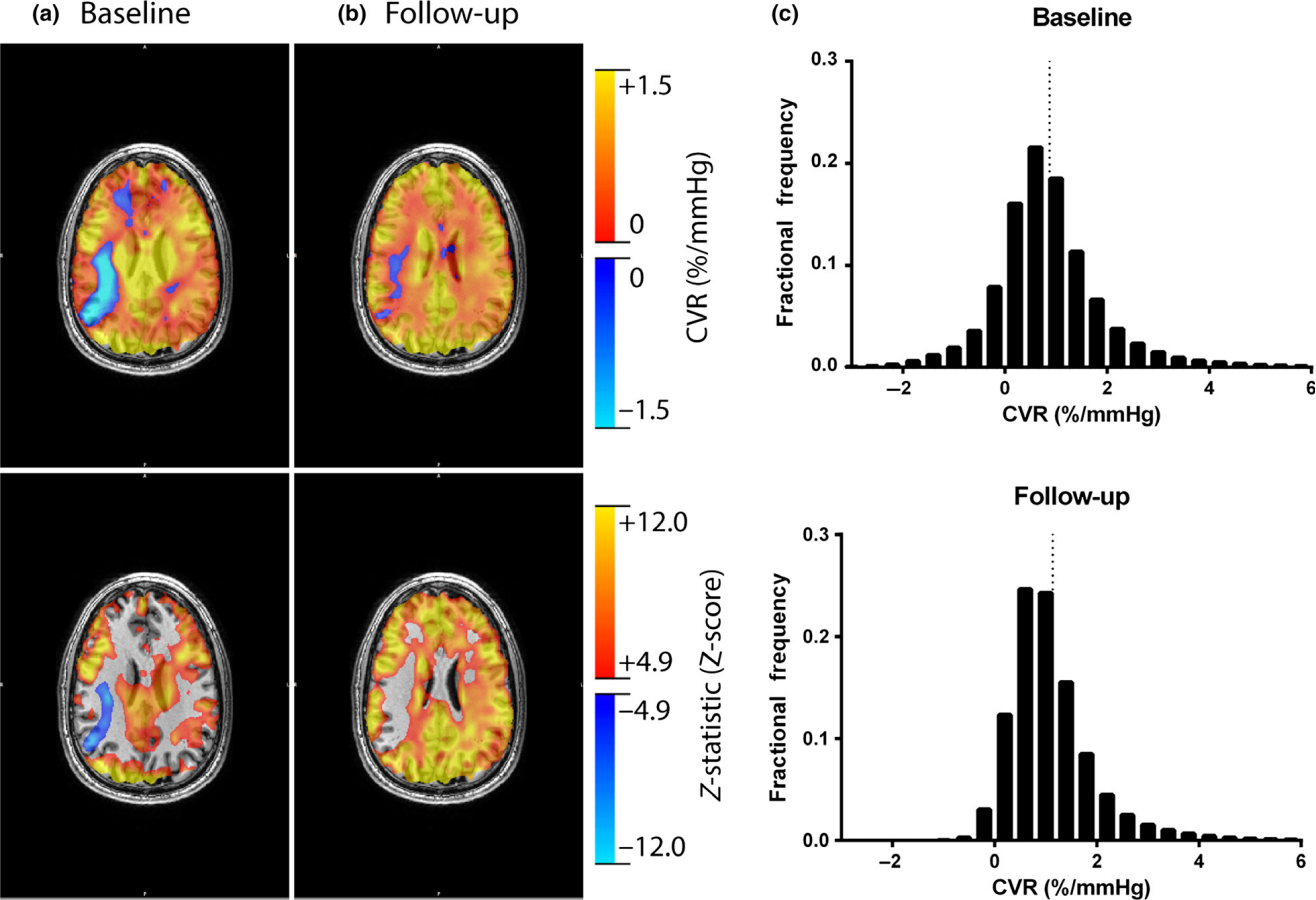
An individual CVR response before and following high‐altitude exposure. Displays cerebral vascular reactivity (CVR) maps and *z*‐statistic maps resel‐corrected to *P* < 0.05 for one individual subject before (A) and following (B) exposure to high altitude. Note the decrease in the degree of negative CVR following exposure to high altitude. The CVR for each voxel was then extracted and a fractional frequency distribution at baseline and follow‐up was generated to illustrate the reduction in voxels responding in the negative direction. The dotted vertical lines mark the mean CVR for each distribution.

Focal areas of negative reactivity were often found in subjects at baseline. Figure 5 shows a sagittal slice CVR map and *z*‐statistic map from before (A) and after high‐altitude exposure in a representative subject (B). Of note are the regions of negative reactivity in the posterior‐right hemisphere and the frontal lobe region. Following exposure to high altitude, the negative reactivity in the frontal lobe had completely normalized and negative reactivity in the posterior‐right hemisphere had decreased in size. Figure 5C displays the fractional frequency distribution of CVR for the same subject at baseline and follow‐up. Note the number of voxels responding in the negative range is substantially reduced at follow‐up and contributes to the rightward shift of the mean CVR (dotted lines in figure).

### Microhemorrhages and white matter hyperintensities

Due to technical problems, microhemorrhages and WMHIs could not be assessed in one subject during his pretest measurement. As a result, only five subjects are reported for microhemorrhages and WMHIs. No microhemorrhages were detected before or after exposure to high altitude in any subject. WMHIs were detected in most subjects at baseline and follow‐up in subcortical structures (*n* = 4) and in some subjects in deep white matter structures (*n* = 2) (see Table 2). In one subject, WMHI were detected in the corpus callosum. WMHI data were heavily skewed as one subject had a total of 37–38 lesions at baseline and follow‐up. As a result WMHI data are reported as the median ± interquartile range (IQR). All WMHI (subcortical and deep) were pooled and found to change very little with 3 weeks of exposure to high altitude (Pre: 3 ± 2; Post: 4 ± 5; median ± IQR).

## Discussion

This is the first study to simultaneously examine the lasting effects of 3 weeks of high‐altitude exposure on cerebral structure and vascular reactivity to CO_2_ in a controlled prospective and longitudinal study design. The major findings from this investigation are threefold. First, exposure to 5050 m for 3 weeks contributes to a consistent and measureable reduction in brain volume that can be attributed to gray matter atrophy. Second, regions of vascular steal (i.e., negative CVR) were detected in otherwise healthy subjects at baseline. Finally, regions of the brain responding with a positive BOLD‐petCO_2_ relationship (i.e., positive CVR) were unaffected by high‐altitude exposure, whereas regions of the brain responding with an inverse BOLD‐petCO_2_ relationship (i.e., negative CVR) improved following high‐altitude exposure.

### Effects of 3 weeks at 5050 m on cerebral structure

We observed a decline in gray matter volume of 2.6 ± 0.4% following 3 weeks at high altitude which exceeds the normal annual rate of decline of 0.83% yr^−1^ (Crivello et al. 2014) and provides confidence in the observed effect. Our results are consistent with findings in nine elite mountain climbers before and following expeditions to even greater altitudes without the use of supplemental oxygen (>6500 m) (Paola et al. 2008). Here, climbers were found to have reduced gray matter density/volume in the left angular gyrus, a region of brain associated with cognitive planning of movement. Although climbers had reduced white matter volume in the left pyramidal tract near the primary and supplementary motor cortex compared to controls, white matter loss was unchanged following the expedition. Consistent with this finding, we also found no change in white matter volume; however, rather than localized effects we found a global reduction in gray matter volume.

We found gray matter tissue loss 1 week following return to sea level compared to baseline. Previous reports suggest that neurotoxicity may be delayed or long‐lasting (Shukitt‐Hale et al. 1996). In one report, rats were exposed to chronic hypoxia (5500 m and 6400 m) for 4 days and were sacrificed at different intervals between 72 and 144 h before conducting a histologic assessment of cell degeneration and death (Shukitt‐Hale et al. 1996). Cell damage was greater the longer the time following exposure to hypoxia. This delay in cell damage suggests that by delaying our post brain scan by 1 week may have contributed to an even greater loss of gray matter tissue compared to other studies which aimed to conduct brain scans as quickly as possible following high‐altitude exposure.

With acute exposure to hypoxia (FiO_2_ = 0.12, a normobaric altitude approximating 5000 m), brain swelling in the white matter is identified between 6 and 16 h and can be exacerbated by exercise (Rupp et al. 2014). Unfortunately, less is known with respect to white matter adaptations following high‐altitude acclimatization and return to sea level. One longitudinal study of 14 mountaineers was unable to detect any gray matter deficits before and following an acute ascent to 6206 m but they did detect a disruption of white matter fiber integrity using tract‐based spatial statistics of white matter fractional anisotropy (Zhang et al. 2012). Although we cannot confirm a disruption of white matter fiber integrity from our MRI measures, our results do suggest that white matter volume remains unchanged following return to sea level after spending 3 weeks at 5050 m. While it is possible that our study is underpowered to detect white matter changes, the 7‐mL change we observed over a 3‐week span at high altitude only modestly exceeds the normal annual rate of decline in white matter (mean ± SEM: 4.54 ± 0.19 mL [Driscoll et al. 2009]). We did not detect any new WMHI or microhemorrhages. The latter is perhaps not surprising as such reports come from subjects who have experienced severe high‐altitude cerebral edema which none of our subjects had (Schommer et al. 2013). Though it is interesting to note that the single subject with the greatest number of WMHI also has had many years of experience in high‐altitude environments.

### Effects of 3 weeks at 5050 m on regional CVR

We examined for the first time the effect that exposure to 5050 m had on CVR throughout different regions of the brain including gray matter, white matter, brainstem, nucleus accumbens, amygdala, caudate, hippocampus, pallidum, putamen, and thalamus (Fig. 3). Across these regions of the brain, CVR varies from its lowest reactivity in white matter tissue to its highest reactivity in gray matter tissue, with the exception of the thalamus, which had a CVR slightly greater than that of pure gray matter tissue. In addition to the variability throughout each region of the brain, we also observed some degree of variation between the hemispheres. The differences in CVR throughout these functional units of the brain are likely attributable to differences in vascularity, pH sensitivity, and metabolic needs. For example, the brainstem and thalamus are highly sensitive to CO_2_, and the maintenance of tissue pH in these regions is important for the control of respiration, and includes chemosensitive neurons in the brainstem and neurons encoding the sensory and affective components of respiration (Chen et al. 1991). Thus, the enhanced CVR in the thalamus may be a function of not only the global CO_2_ sensitivity of the vasculature in this region but also the result of increased metabolism linked to the role of the thalamus in relaying information pertaining to respiratory sensations and respiratory drive (Chen et al. 1991). Interestingly, the brainstem and thalamus are believed to be phylogenetically older regions of the brain and hence may be more sensitive to hypoxia (Binks et al. 2008).

The spatial heterogeneity of CVR remained following exposure to high altitude; however, most regions of the brain had a slight increase in CVR reaching significance in the brainstem, hippocampus, and thalamus (Fig. 3). Our findings are in contrast to Villien et al. (2013) who used arterial spin labeling MRI to assess CVR in the MCA territory in healthy humans before and following 6 days at 4350 m. In addition, they made measurements of CVR while at high altitude using transcranial Doppler ultrasound of the MCA. Similar to their high‐altitude measurements they found that CVR was reduced following return to sea level. However, measurements were made within 6 h following return to sea level where significant ventilatory responsiveness, hypocapnia, and hematologic adaptations make comparison to our study difficult. Thus, we suggest that 1‐week following return to sea level might be an appropriate time point for posttesting in the MRI since arterial PCO_2_ has normalized. Furthermore, the use of end‐tidal forcing provides a methodology for controlling arterial blood gases independent of ventilation such that the stimulus for cerebral blood flow reactivity is consistent (Fierstra et al. 2013). Such devices reduce the variability in the BOLD signal and cerebral blood flow by reducing the breath‐by‐breath fluctuations in arterial PO_2_ and PCO_2_ (Chang and Glover 2009), reduce the distortion of CVR by the CO_2_‐induced changes in petO_2_ (Prisman et al. 2007), and can protect against the variability in arterial PCO_2_ between subjects due to differences in chemosensitivity.

### The importance of delineating negative and positive CVR

The frequency distribution of CVR in healthy subjects illustrates a normal distribution including a significant proportion of negative voxels (Sobczyk et al. 2014). The negative voxels represent regions of the brain with an inverse response to CO_2_ and suggests a reduction in brain blood flow despite the presence of a global dilator stimulus. On the basis of this finding, we felt it necessary to dichotomize each anatomical region of the brain into regions of positive and negative CVR at baseline. In doing so, we were able to determine if a change in negative CVR was directly responsible for the increase in CVR we observed in the brainstem, hippocampus, and thalamus. Figure 4 illustrates that regions of positive CVR were unaffected by 3 weeks at 5050 m, whereas the regions of negative CVR gained a significant degree of positive reactivity and contributed entirely to each regions increase in CVR.

### Implications of negative vascular reactivity in otherwise healthy volunteers

While there is evidence to suggest that reduced CVR and vascular steal is associated with enhanced risk of stroke (Webster et al. 1995), is negative CVR detected in healthy individuals the result of vascular steal? We studied a group of young subjects with no history of cardiovascular and respiratory disease. Voxels of negative CVR were present in all anatomical structures including gray matter, white matter, brainstem, nucleus accumbens, amygdala, caudate, hippocampus, pallidum, putamen, and thalamus. Interestingly, the magnitude of negative CVR was greatest in the brainstem, an area with important chemosensitive neurons and responsible for cardiovascular and respiratory control. As described above, we suspect that small clusters of negative CVR, which are heterogeneously scattered throughout the brain, are linked to the vascularity or capillary density of a voxel. In this context, negative CVR may reflect a capillary bed maximally dilated to maintain adequate perfusion and is normally operating at its vascular reserve (Sobczyk et al. 2014). However, following global hypoxia this region becomes stressed and is able to normalize its response through the process of angiogenesis and microvascular hyperplasia. An increase in the BOLD response could in part be explained by having more vessel volume in a given voxel and thus is some evidence for angiogenesis. The mechanisms explaining our observations are currently unknown, but likely involve the complex interplay between hypoxia‐induced angiogenesis (LaManna et al. 1992), stimulation of neuronal pathways, microvascular hyperplasia (Harik et al. 1995), release of adenosine, endothelium‐derived nitric oxide and a variety of autocoids and cytokines (reviewed in: Ainslie and Ogoh 2010). These possibilities need to be explored in future studies.

In contrast to voxels of negative CVR scattered throughout the brain are focal regions of negative CVR. Focal regions of negative CVR are more likely impacted by downstream vessel disease impairing blood flow delivery to a much larger capillary network (Sobczyk et al. 2014). Regions of negative CVR in normal subjects are typically found in regions of deep white matter (Mandell et al. 2008) and thus it was surprising to observe significant regions of negative CVR in otherwise healthy subjects outside of deep white matter. Figure 5 illustrates a large focal region of negative CVR in the right hemisphere in one subject at baseline and follow‐up. While negative CVR could be an artifact of a poor correlation, this seems unlikely since the *z*‐statistic of voxels in these regions reach statistical significance and the CVR stimulus (i.e. petCO_2_) was identical for pre and post tests (illustrated in Fig. 1). If CVR is plotted as a fractional frequency distribution (see Fig. 5C) then it is possible to observe how the distribution of CVR across the whole brain is affected by high‐altitude exposure. At baseline prior to altitude exposure, the CVR is normally distributed with the majority of CVR being positive. However, following 3 weeks of altitude acclimatization and return to sea level, the proportion of negative CVR scores is dramatically reduced with a concomitant increase in the frequency of positive CVR. This reduction in negative CVR leads to an increase in the mean CVR. However, considering the results from Figure 4, the increase in mean CVR seems to be the result of a functional change in regions of negative CVR rather than those regions of the brain, which already responded in the expected positive manner.

### Methodological considerations

Dehydration could be a confounder for the volumetric and morphometric analyses conducted in this study. It has previously been demonstrated using voxel‐based morphometry and longitudinal brain‐change detection algorithm's (i.e., SIENA) that gray and white matter volume measurements are associated with hydration status (Streitbürger et al. 2012). For example, dehydration led to significant reductions in gray and white matter when using voxel‐based morphometry. Using this approach, significant reductions in gray and white matter were found in the left inferior orbito‐frontal region, and in the extranuclear region. Interestingly, we did not detect any focal changes in gray or white matter using a voxel‐based morphometry approach. Dehydration may have been a factor while at high altitude, but at sea level it is likely that hydration status was close to normal (Singh et al. 1990).

We used BOLD as a surrogate for cerebral blood flow, which is well accepted (Hoge et al. 1999); however, CVR obtained from BOLD is not comparable in magnitude to CVR obtained by arterial spin labeling or Doppler ultrasound. In addition, the blood pressure response to CO_2_ is an important confounding variable when assessing CVR and we were unable to acquire blood pressure inside the MRI and therefore cannot account for its effects on CVR at follow‐up. However, Smirl et al. (2014) assessed cerebral pressure‐flow relationships before and following high‐altitude exposure in a set of subjects from the same high‐altitude expedition. Two weeks after return to sea level hemodynamic and cardiovascular measures had returned to baseline levels including spontaneous metrics of cerebral pressure‐flow relationships. Such findings indicate that following high‐altitude exposure the cerebral autoregulation is normal.

This study is novel because it simultaneously addresses the issue of brain volume and regional CVR before and following 3 weeks of high‐altitude acclimatization in the Himalayas of Nepal in a small sample of healthy humans (*n* = 6). The feasibility issues (i.e., cost, logistical) involved in performing field research of this nature often involve a trade‐off in statistical power due to smaller sample sizes. As argued in detail elsewhere (Ploutz‐Snyder et al. 2014) the value of small sample size research is indeed appropriate when larger sample sizes are not feasible. We acknowledge that our sample size limits our ability to detect every region specific differences in brain volume, WMHI and microhemorrhages and the related relationships with CVR. However, we do present compelling data in both mean and individual forms that clearly demonstrates a consistent effect on total brain and gray matter volume. More importantly, for the first time, we illustrate a clear and significant effect of 3 weeks of high‐altitude exposure on regions of negative CVR, which is consistent across all subjects and independent of regions of positive CVR. These results are unlikely due to methodological error as methodology and equipment were identical at baseline and follow‐up. Others have reported the between‐day coefficient of variation for CVR to be 6.8 ± 1.2% and 9.9 ± 1.8% for gray and white matter, respectively (Kassner et al. 2010). In addition, the percent brain volume change normally has a 0.2% overall error rate (Smith et al. 2007). Our principle effects are outside the day‐to‐day measurement error. While our data does not include a low altitude time control, and it is possible that cardiovascular fitness may have improved during the 8‐day trek to high altitude, it seems unlikely that this would have persisted during the 3 weeks at the high‐altitude research laboratory where participants led a primarily sedentary lifestyle conducting and participating in experiments.

## Conclusion

We found that 1‐week following return to sea level, brain volume was significantly reduced and attributed to a loss of gray matter tissue. In addition, our results highlight the need to dichotomize regional CVR measurements into positive and negative responding units. By doing so we found that regions of positive CVR were not impacted by high‐altitude exposure, but regions of negative CVR significantly gained functionality.

## Conflict of Interest

None declared.
